# Older adults’ perceptions of government handling of COVID-19: Predictors of protective behaviors from lockdown to post-lockdown

**DOI:** 10.1371/journal.pone.0263039

**Published:** 2022-02-02

**Authors:** Savannah Kiah Hui Siew, Jonathan Louis Chia, Rathi Mahendran, Junhong Yu

**Affiliations:** 1 Department of Psychological Medicine, Yong Loo Lin School of Medicine, National University of Singapore, Singapore, Singapore; 2 Psychology, School of Social Sciences, Nanyang Technological University, Singapore, Singapore; 3 School of Social Sciences, Singapore Management University, Singapore, Singapore; The University of Hong Kong, HONG KONG

## Abstract

**Background:**

Distrust, and more broadly, public perception of government’s handling of a crisis, has been a widely studied topic within health crisis research and suggests that these perceptions are significantly associated with the behavior of its citizens.

**Purpose:**

To understand which aspects of the public’s perception of government handling of the COVID-19 pandemic predicted engagement of protective behaviors among older adults, who are the most vulnerable to COVID-19.

**Methods:**

Participants were recruited from an ongoing biopsychosocial study on aging amongst community-dwelling older adults. There were two rounds of data collection, during the national lockdown and post-lockdown. The average length of follow-up was 5.88 months. N = 421 completed the first round of data collection and N = 318 subsequently completed the second round of questionnaires.

**Results:**

During the lockdown, perceptions that pandemic-related measures in place were sufficient, effective, timely, provided a sense of safety, important information was easily accessible, and government handling of the pandemic could be trusted, were found to significantly predict engagement in protective behaviors. During post-lockdown, only perceptions that measures in place were sufficient, provided a sense of safety, and important information was easily accessible, remained significant predictors. The perception that COVID-19 measures were clear and easy to understand now became a significant predictor.

**Conclusions:**

Public perceptions of government handling of the pandemic predicted engagement in protective behaviors but were less important during post-lockdown. To effectively engage older adults in protective behavior, our findings suggest for pandemic-related information to be accessible, introducing timely safety measures, and having easy-to-understand instructions for nuanced measures.

## Introduction

The current COVID-19 pandemic has led to major worldwide economic and social disruptions, and unprecedented mortality rates. As of April 2021, there were around 153 million confirmed cases, and a reported death toll of around three million [1]. Much of the social disruptions have resulted from drastic safe distancing measures implemented to curb the virus spread [2]. To deal with surges of COVID-19 cases, numerous countries implemented lockdowns and other disparate measures to curb local transmissions.

The Swedish government for example, espoused herd immunity developed through community infections, before vaccines were developed. This approach was criticized, given the high fatality of COVID-19 infection [3] and relative low efficacy of the approach [4]. The national pandemic response in the United States was heavily criticized when in the initial weeks critical to establishing an effective outbreak response, the then administration denied the danger of the virus, was unclear about the efficacy of tested public health guidelines, and promoted untested drugs and medical procedures [5]. Further differing decisions by individual American states on social distancing measures, ranging from lifting of all social distancing measures to slower stepwise adoption, led to public confusion and distrust [6, 7].

**Fig 1:**
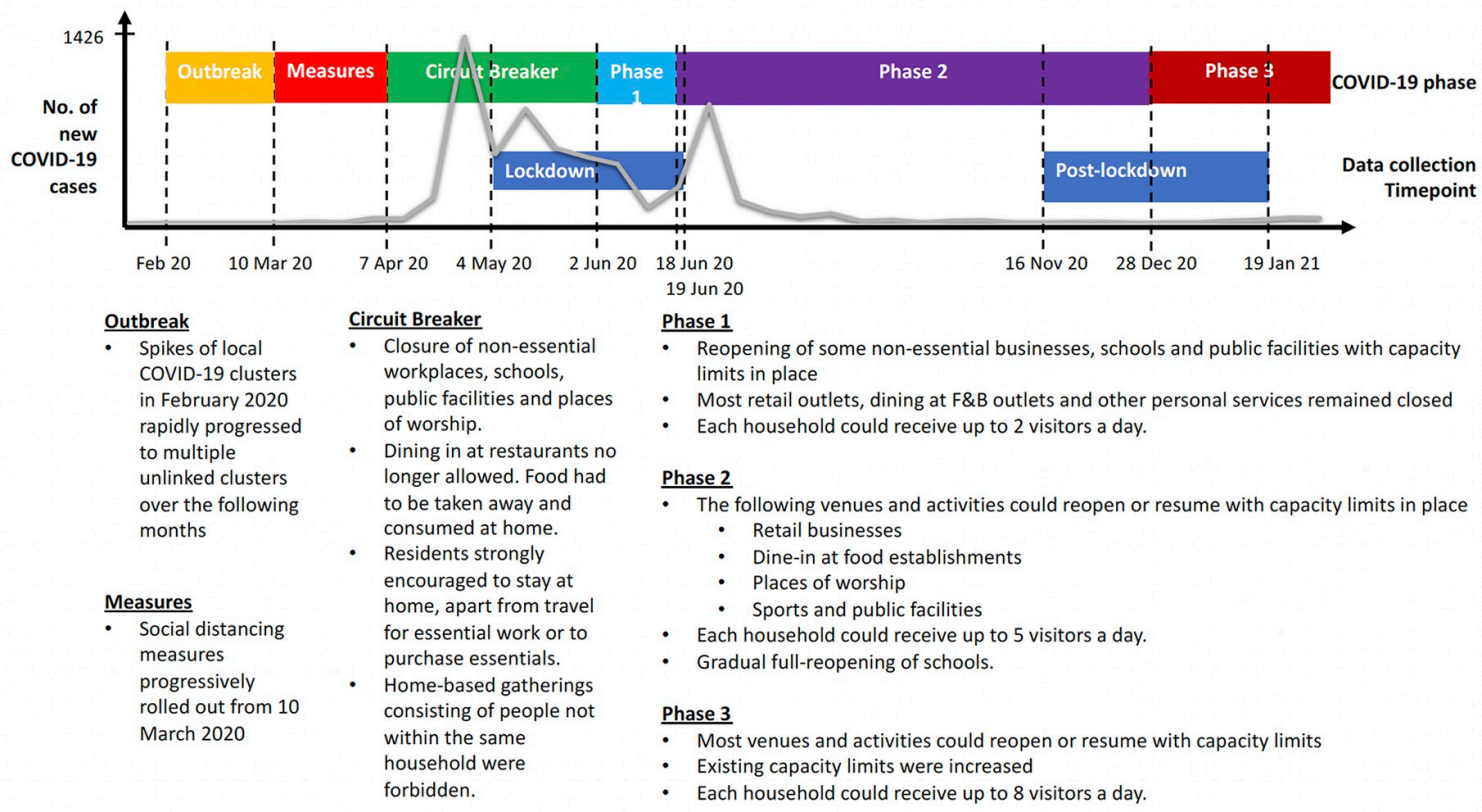
COVID-19 and its lockdown situation in Singapore.

In Japan, one of the first countries to have a COVID-19 case outside of China, the state of emergency was declared as early as 28 February 2020. However, this state of emergency differed starkly from the lockdowns implemented in other countries because there was no enforceability, and it was dependent on the citizen’s voluntary modification of their behavior [8]. Similarly, South Korea did not implement a national lockdown but instead depended heavily on intensive testing, contact tracing and isolation [9]. Taiwan leaned into border controls early and conducted proactive testings because of their proximity to mainland China and a large number of flights between the two places [10]. The unfolding of the COVID-19 situation here in Singapore is presented in detail in Fig 1.

Distrust, and more broadly, public perception of government’s handling of a crisis, has been a widely studied topic within health crisis research and suggests that public perceptions of government is significantly associated with the behavior of its citizens. The literature on protective behavior during the outbreak of Severe Acute Respiratory Syndrome (SARS) reveals that individuals who believed authorities were open with their communication, were more likely to engage in protective behaviors such as covering their mouth when coughing or sneezing, washing their hands with soap, and wearing masks [11, 12]. Conversely, inconsistent information from authorities was associated with doubt over the efficacy of protective behavior and consequently, quarantine compliance [13]. This relationship between protective behavior and government perception was again observed during the H1N1 outbreak; high level of trust in authorities and satisfaction with the communications received about the disease were associated with compliance and protective behavior [14]. Similar patterns of protective behavior and government perception have been reported even during periods absent of an epidemic or pandemic [15].

Singapore, among other nations such as Taiwan and New Zealand, had been touted globally as a nation effective in its management of the pandemic. Singapore’s success had been credited to early response, extensive testing, contact tracing, citizen’s knowledge and awareness of the virus, and social distancing measures, against the backdrop of sociopolitical success [16–20]. Adoption of many COVID-19 safety measures does not come easily without sociopolitical success. Drastic measures such as a national lockdown, and even less drastic measures such as mandatory mask-wearing, have been subjected to scrutiny and pushback [18]. Resistance between the government and its citizens can result in delayed widespread adoption of these safety measures [21], a delay which might make the difference when it comes to infectious disease containment. An understanding and insight of how the Singapore government and its handling of the pandemic is perceived by its citizens, in relation to protective behavior, could serve as a guide to future pandemic responsiveness. The COVID-19 has disproportionately taken the lives of older adults because of medical co-morbidity, increased risks of complications and weaker immune systems [22].

The study aim was to focus on the effects on protective behaviors among older adults as they faced the greatest vulnerability for illness severity and mortality during the COVID-19 pandemic. An exploratory study was thus conducted on various factors of the public’s perception, to determine which aspects of the government’s handling of the pandemic predicted engagement of protective behaviors amongst older adults.

## Materials and methods

### Participants and procedures

Participants were recruited from the ongoing Community Health and Intergenerational (CHI) study investigating biopsychosocial factors of aging amongst community-dwelling older adults. Details regarding the recruitment, inclusion criteria, and study procedures have been described by Lee et al [23]. All participants from the CHI study, except those diagnosed with dementia and those who could not read or write in English or Chinese, were contacted by phone during Singapore’s COVID-19 lockdown period.

N = 421 gave written informed consent and completed the study questionnaires between 4^th^ May 2020 and 19^th^ June 2020. In a subsequent invitation to respond to the questionnaires in a second round of data collection between 16^th^ November 2020 to 19^th^ January 2021, N = 318 participants responded. Fig 1 shows how the data-collection timepoints coincided with the phases of COVID-19 related restrictions in Singapore. Participants were given the choice to complete the questionnaire online or on a hard copy mailed to them. The average length of follow-up was 5.90 months (SD = 0.43). A ten-dollar remuneration was given to participants upon completion of each set of questionnaires. The study had ethics approval from the National University of Singapore’s Institutional Review Board (Ref No. S-20-118E). The demographic characteristics of the sample are shown in Table 1.

**Table 1:** Descriptive statistics of participants.

	Baseline characteristic	Follow-up characteristic
	Mean (S.D.)/Frequency	Mean (S.D.)/Frequency
N	421	318
Age	69.09 (5.45)	69.17 (5.46)
Gender		
Female	275	207
Male	146	111
Housing		
1–2 room HDB	17	12
3 room HDB	24	16
4–5 room HDB	130	98
Executive/Maisonette	52	41
Private Apartment/Condominium	97	73
Landed Housing	99	76
Ethnicity		
Chinese	402	306
Malay	2	2
Indian	14	8
Others	2	1
Living Arrangements		
Alone	63	49
With others	350	265
Education (years)	13.55 (3.83)	13.48 (3.76)
CRPB Total Score	17.17 (2.41)	16.66 (2.28)

HDB = Housing Development Board, which refers to public housing in Singapore; CRPB = COVID-19 Risky and Protective Behaviors.

### Measures

#### COVID-19 risky and protective behaviors

Protective behaviors relating to COVID-19 were measured using a 12-item Covid-19 Risky and Protective Behaviors (CRPB) questionnaire (see S1 Appendix), derived from guidelines provided by the World Health Organization and local health authorities in Singapore [24, 25]. The frequency of risky and protective behavior engagement over the past one week was assessed using a five-point Likert scale, with option 1 representing “Never” and option 5 representing “All the Time”. A total score was derived by adding up the scores on each question such that higher scores represent higher engagement in protective behaviors.

Eight items were removed as they were no longer meaningful measures of protective behaviors since they have become mandatory by law (i.e., wearing of masks and prohibition of social gatherings) or were not specific to the COVID-19 context (i.e., general hygiene-related items). The remaining 4 items, namely: (1) avoid touching face, (2) social distancing, (3) avoid crowded places and (4) avoid physical contact were more specific and salient to the current COVID-19 context. This 4-item-abridged version of the CRPB was internally consistent and reliably assessed protective behavior across both time points, as indicated by our structural equation modeling results (see S1 File). Briefly, these results showed that the final longitudinally invariant model, where unstandardized item factor loadings are held constant across time, fitted well to a single factor model. In the regression analysis below, we only included items from this SEM model in the calculation of the total CRPB score.

#### Singapore’s handling of COVID-19

Perception of overall government handling was assessed through eight items on the self-developed Singapore’s Handling of Covid-19 (SHC) questionnaire (see S2 Appendix). Four questions were focused on the government’s handling of COVID-19 through the measures they implemented. These questions assessed whether the measures were sufficient, effective, whether the measures made people feel safe, and the timeliness of the measures being rolled out. These were targeted at the different facets of the success of the measures implemented by the government. Two questions were related to how the government dealt with communicating information surrounding COVID-19, whether important information was easily accessible, and whether the information was clear and easy to understand. The efficacy of the measures hinges on the ability of the government to ensure that the relevant information reaches its intended audience and is understood by them. The other two questions were to gain a general understanding of the perception of the government during the COVID-19 crisis by asking participants if they trusted the government and whether the government was handling the situation well. Responses were recorded using a five-point Likert scale, with option 1 representing “Strongly Disagree” and option 5 representing “Strongly Agree”.

Covariates measured were age, gender, and education; education was measured using years of schooling. As participants had previously participated in a cohort aging study, covariates data had been previously collected and was used for this study.

### Statistical analysis

All analyses were performed in R 4.0.3. The R code for executing the analyses below is available at https://osf.io/h3un8/?view_only=ac5500d5ae16435c943a6c247f4ad9a7.

#### Comparing means across time

Paired sample Wilcoxon Test was conducted to determine the statistical significance of the difference in means during the lockdown and post-lockdown period for individual SHC items and the CRPB total score. Statistical significance was set at *p* < 0.05.

#### Item-level regression analysis

Item-level linear regression was conducted to examine the predictive effect of individual SHC items against the CRPB total score at time point 1 and time point 2. The covariates of age, sex, and education were controlled for during regression analysis. Statistical significance was set at *p* < 0.05. Although it may seem unusual to conduct linear regression using an ordinal predictor, experiments with real and simulated data have shown that parametric statistics can still be used with Likert scale data as it remains robust despite violations of the statistical tests’ assumptions [26].

## Results

### Comparing means across time

**Fig 2:**
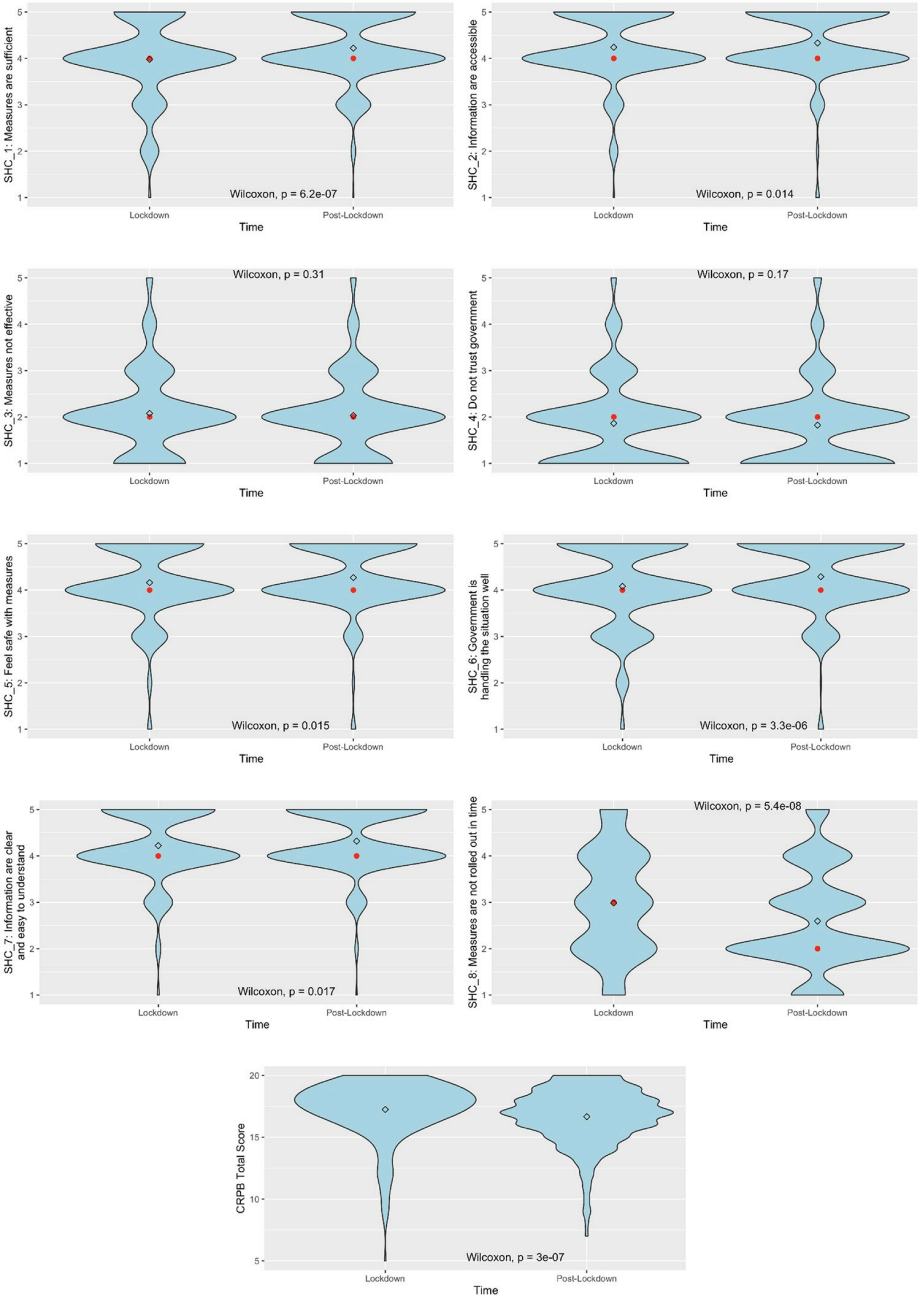
Violin plots of descriptive statistics of measures. The red dot represents the median while the diamond represents the mean. Paired sample Wilcoxon test was used to compare the means across time and the statistical significance was determined by its p-value, reported in the figure. SHC, Singapore’s Handling of COVID-19; CRPB, COVID-19 Risky and Protective Behaviors.

Fig 2 shows the descriptive statistics of the changes across time of both SHC individual items and CRPB total score. Public perceptions of the government’s handling of COVID-19 generally had significant improvements across time, with small to moderate effect sizes ranging from .08 ≤ *r* ≤ .32. Perceptions that measures were not effective had the smallest change over time while perceptions that measures were not rolled out in time had the largest change over time. Our results also showed that older adults tend to engage in significantly fewer protective behaviors over time with a moderate effect size (*r* = .31).

### Item-level regression analysis

**Fig 3:**
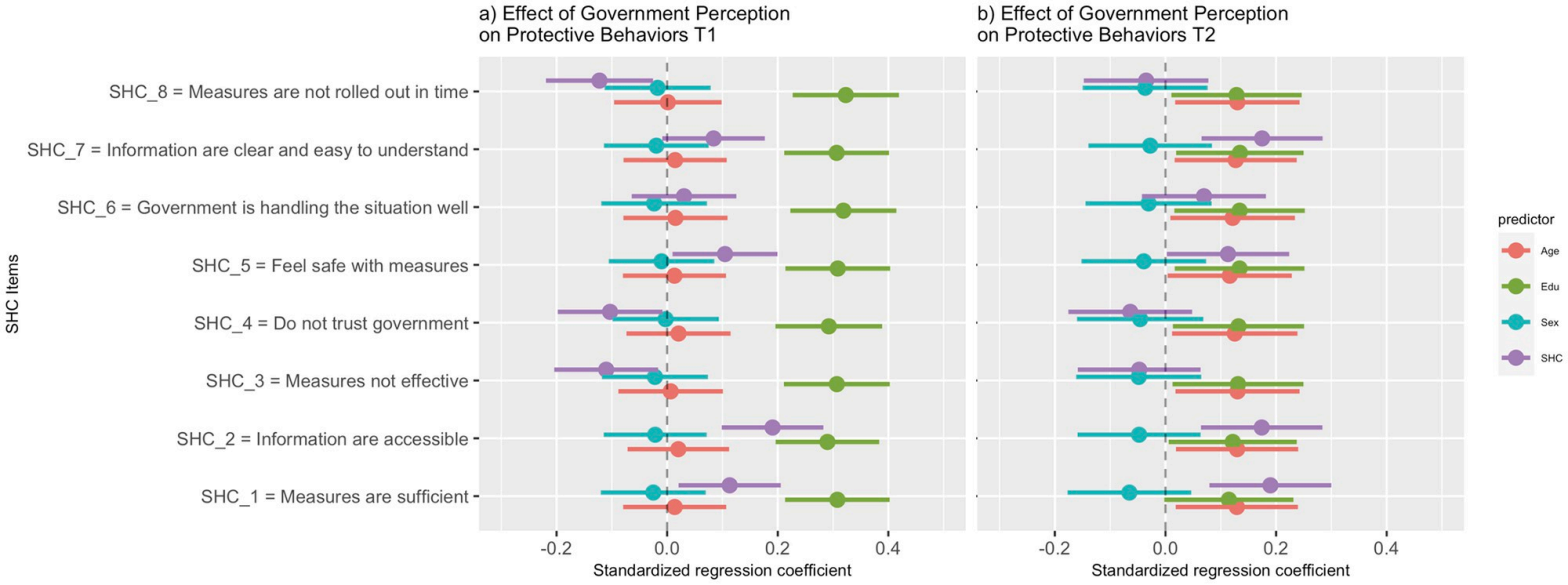
Forest plot of standardized regression coefficients of SHC items and covariates on CRPB total score. The standardized regression coefficients are represented by the dots within their respective lines that corresponds to its 95% confidence intervals. If age, education, or SHC predictors fall on the left side of the figure’s 0 line, it means that an increase in those variables predicts a decrease in protective behaviors. If the sex predictor falls on the left side, it means that males tend to engage in less protective behaviors than females in that SHC model. The opposite is true if it falls on the right side. SHC, Singapore’s Handling of COVID-19. a) T1, during lockdown, b) T2, during post-lockdown.

The results of the regression analysis during the lockdown and post-lockdown periods can be seen in Fig 3.

#### During lockdown (Timepoint 1)

Most perceptions related to the government’s handling of COVID-19 were significant predictors of engagement in protective behaviors. The following factors significantly predicted more engagement in protective behaviors. When participants feel that COVID-19 measures in place are sufficient, effective, make them feel safe, and are rolled out in time to deal with the fast-changing situation, important information is easily accessible, and they trust the government in handling the situation. However, the following factors did not significantly predict more engagement in protective behaviors. When participants feel that the government is handling the COVID-19 situation well and information on measures are clear and easy to understand.

The age and sex of the participants were not significant predictors of engagement in protective behaviors in any of the regression models. On the other hand, years of education significantly predicted higher engagement in protective behaviors in all the regression models.

#### Post-lockdown (Timepoint 2)

Only half of the perceptions related to the government’s handling of COVID-19 were now significant predictors of engagement in protective behaviors during the post-lockdown period. Participant’s perception that measures put in place are sufficient, important information is easily accessible, and they feel safe with the measures remained significant predictors of protective behavior. Participant’s perception that COVID-19 measures are clear and easy to understand was now a significant predictor too.

Sex was still not a significant predictor of engaging in protective behaviors in any of the regression models. However, the age of participants now significantly predicts engagement in protective behaviors in most regression models; those who are older have higher engagement in protective behaviors. Years of education remain a significant predictor of protective behavior except in one model.

## Discussion

The results showed that public perceptions of the government’s effort during the pandemic predicted engagement in protective behaviors but were less important predictors during the post-lockdown period. Moreover, public perceptions have improved over time with the improved COVID-19 situation. Education was a predictive factor of higher engagement in protective behaviors, but the sex of the participant was not, regardless of the time point. Age was not a significant predictor during the lockdown period, but it became significant during the post-lockdown period.

During the lockdown period, public perception of the COVID-19 measures-related information accessibility is an important aspect of the government’s effort because it accounts for the largest proportion of the variance, among other predictors, in protective behaviors. This relationship can be attributed to a consistent source of information that is associated with less doubt over the credibility of available information and this, in turn, affects their risk perception and subsequent compliance behaviors, as shown by a study during the SARS outbreak [13]. Their research surfaced a need for public health communications to be credible because it can affect the perceptions of risk during this crisis and in turn significantly increase compliance to quarantine protocols which can help contain pandemic outbreaks. Our finding is consistent with previous associations found between satisfaction with disease communications from authorities and protective behavior [14]. This review paper presented the finding that beliefs that authorities were open with their communication were associated with protective behaviors such as washing hands after sneezing and wearing a mask. On the other hand, inconsistent information from the authorities was associated with lower compliance with quarantine because of doubts regarding the credibility of the given information. Public perception that measures were rolled out in time is the second most important aspect, probably because of the urgent and fast-changing nature of the pandemic especially during the lockdown period, which saw our highest daily total cases topped day after day. However, years of education still accounted for a larger proportion of variance explained in protective behavior than any of the public perceptions of the government. This reiterates the findings of local studies where those with higher education tended to engage in more protective health behaviors during the earlier part of COVID-19 [27] and past pandemics [11, 14].

As the pandemic drew longer and the country moved into a post-lockdown period, there are some changes observed in the relationship between public perception of the government and engagement in protective behaviors. Participant’s perception that COVID-19 measures were clear and easy to understand now became a significant predictor. This is presumably because the measures in Singapore were constantly changing as the country transitioned after a strict lockdown period termed "circuit breaker" to a less restrictive period. If measures were not clear and easy to understand, it can cause confusion and frustration among citizens leading to a breakdown of adherence to safety guidelines and protective behaviors. The perception that measures were rolled out in time to deal with the fast-changing nature of the pandemic was no longer a significant predictor during the post-lockdown period. While many countries around the world had a second resurgence of COVID-19 infections and faced a very volatile situation, Singapore had a much more stable COVID-19 situation after the first peak.

Generally, public perception of the government seems to matter less over time with regard to engagement in protective behaviors. There is also a shift in importance from the effectiveness of measures to communication strategies during the post-lockdown period. Messages from the government during a pandemic should be consistent and coordinated as this will raise the level of trust in the sources of information which is associated with engagement in protective behaviors [28]. These findings could benefit other countries when their situations improve too.

There has been a long-standing interest in the relationship between public perception of government and citizen behavior. The current COVID-19 pandemic has certainly highlighted the importance of research in this area. While research has established the relationship between government perception and protective behavior to some extent, these studies have mainly centered around government trust [15, 27, 29–32]. However, government trust as a broad variable does not always relate directly to protective behaviors [30]. Government handling of the health crises involves other related domains besides trust. These domains include information communication, timeliness of response, and efficacy of specific measures. To better understand the nuanced relationship between government perception and protective behavior, it is important to tease out how these different domains affect protective behavior.

As evidenced by our results, the role of the public’s perception of the government, which does not only include government trust, is fundamental in shaping engagement in protective behaviors, a crucial tool for controlling the current pandemic by reducing the chance of transmissions. Our research points to the following domains as the more important ones:

*(1) Making information accessible*. Information regarding the pandemic should be communicated through channels and platforms easily accessible to all members of society. More importantly, perceptions are often influenced by the information received and thus, it is crucial where these sources of information come from. Both traditional and social media play an outsized role in shaping the information people receive and this, in turn, influences their perception. News, be it factual or fake, travel fast today through the internet. The latest information on the pandemic must be promptly communicated and accessible through official channels. Within our sample, official sources such as health officials, and television and newspaper, which are state-controlled, are the main sources of information (see S1 Table). Thus, the relationship observed between government perception and protective behaviors could be attributed to this consistent and reliable source of information. It is important to acknowledge that current reliance on technological communication channels may exclude those with socio-economic difficulties or those with lower education because they might lack access to smartphones or the internet. The importance of education is alluded to in our results as a significant predictor of protective behaviors. Thus, it is imperative that those who lack access to technology are not systemically left out of receiving crucial information. For example, advertising spaces in public transports such as trains and buses, or bulletin boards managed by the town council in public housing areas can be used to display pandemic-related information during this period.
*(2) Measures rolled out in a timely manner*. Although this suggestion might seem obvious, it is important to understand the speed at which global news travels. Measures rolled out may seem timely to local health officials, however, citizens may judge the timeliness of these measures with respect to other countries. As such, it is important to understand the expectation and perception of citizens regarding the timeliness of measures implemented, especially during a pandemic of a global nature.
*(3) Easy-to-understand instructions for nuanced measures*. The message during the lockdown is clear—stay at home and avoid going out. During post-lockdown, policies and measures become more varied. For example, post-lockdown measures include rules on the maximum number of people allowed in a group and the resumption of certain businesses or public activities and not others. Such nuanced measures must be clearly communicated to maintain citizen engagement in protective behavior and reduce confusion. Maintaining citizen engagement in protective behavior during the post-lockdown period is important in preventing a resurgence of cases and the need for another lockdown.

The study findings are subjected to some limitations. Firstly, the socio-cultural context of Singapore’s population’s relationship with the government should be taken into consideration. The government has been traditionally ruled by one party for a long time and their good track record of effectively handling past crises is demonstrated. Moreover, there is a strong emphasis on deterrent measures, such as fines, to ensure compliance and this has resulted in relatively high compliance with governmental guidelines. Singapore is a collectivistic society and collectivism tends to predict engagement in protective behaviors during COVID-19 [33] which could limit the generalizability to individualistic societies. However, we still detected changes within the same population from the lockdown to the post-lockdown period which is possibly generalizable to other countries.

## Supporting information

S1 Appendix COVID-19 risky and protective behavior questionnaire. *Note*. Bolded items are those included in our analyses.
(DOCX)

S2 Appendix Singapore’s handling of COVID-19 questionnaire. (DOCX)

S1 File Structural equation modelling results. (DOCX)

S1 Table Participants’ sources of COVID-19 related information. *Note*. Participants were asked “Please indicate how much information about COVD-19 you have received from the following sources by selecting the appropriate option.” on a 5-point scale, “none”, “little”, “some”, “much”, and “very much”.
(DOCX)
