# Supplementary material for: Older adults’ perceptions of government handling of COVID-19: Predictors of protective behaviors from lockdown to post-lockdown
Source: PLoS One. 2022 Feb 2;17(2):e0263039. doi: 10.1371/journal.pone.0263039 (PMC8809562; doi:10.1371/journal.pone.0263039)
Supplement: S1 Appendix — Note. Bolded items are those included in our analyses. (DOCX) [file pone.0263039.s001.docx]

| 1. Fail to wash your hands with soap and water or use an alcohol-based hand sanitizer before and after meals, after coughing/sneezing/blowing your nose or after visiting the restroom. |
| --- |
| 1. **Avoid touching your face.** |
| 1. Clean frequently touched surfaces and objects such as table tops and keyboards. |
| 1. Wear a mask when you are outside. |
| 1. Cover your mouth and nose when you cough or sneeze with flexed elbow or tissue and dispose the used tissue immediately. |
| 1. **Practice social distancing of at least 1 metre apart.** |
| 1. Host or attend social gatherings with 5 or more people (e.g., religious, private or work-related gatherings). |
| 1. **Avoid crowded places.** |
| 1. Keep yourself updated with verified news regarding COVID-19. |
| 1. Take your temperature twice a day. |
| 1. **Avoid unnecessary physical contact (e.g., hand-shaking).** |
| 1. Increase ventilation for enclosed spaces by opening the windows as much as possible. |
