# Supplementary material for: Older adults’ perceptions of government handling of COVID-19: Predictors of protective behaviors from lockdown to post-lockdown
Source: PLoS One. 2022 Feb 2;17(2):e0263039. doi: 10.1371/journal.pone.0263039 (PMC8809562; doi:10.1371/journal.pone.0263039)
Supplement: S2 Appendix — (DOCX) [file pone.0263039.s002.docx]

|  | The measures put in place for COVID-19 are sufficient. |
| --- | --- |
|  | Important information regarding COVID-19 are easily accessible. |
|  | The measures put in place for COVID-19 are not effective. |
|  | I do not trust the government in handling the COVID-19 situation. |
|  | I feel safe with the measures put in place for COVID-19. |
|  | The government is handling the COVID-19 situation well. |
|  | Information on COVID-19 measures are clear and easy to understand. |
|  | The measures for COVID-19 are not rolled out in time to deal with the fast-changing situation. |
