# Supplementary material for: Older adults’ perceptions of government handling of COVID-19: Predictors of protective behaviors from lockdown to post-lockdown
Source: PLoS One. 2022 Feb 2;17(2):e0263039. doi: 10.1371/journal.pone.0263039 (PMC8809562; doi:10.1371/journal.pone.0263039)
Supplement: S1 File — (DOCX) [file pone.0263039.s003.docx]

Structural equation modeling (SEM) was carried out with the R package lavaan [1]. SEM was used to validate the CRPB scale developed for this study by removing items that were unreliable till a good model fit was reached. Parameters estimate for the SEM were computed using the robust maximum likelihood estimator. Root Mean Square Error of Approximation (RMSEA), Comparative Fit Index (CFI) and Standardized Root Mean square Residual (SRMR) were used to assess model fit. CFI values greater than 0.95 were considered a good fit [2], while RMSEA and SRMR values less than 0.06 and 0.08 respectively were considered relatively good fit [3]. Statistical significance was set at *p* < 0.05.

The 4-item CRPB model with items 2, 6, 8 and 11 included had good fit indices (*χ*^2^(18) = 34.37, *p* = .011, CFI = .956, RMSEA = .046, SRMR = .053) and was chosen as the model used in the study. The results of the SEM with item loadings are presented in the figure below.


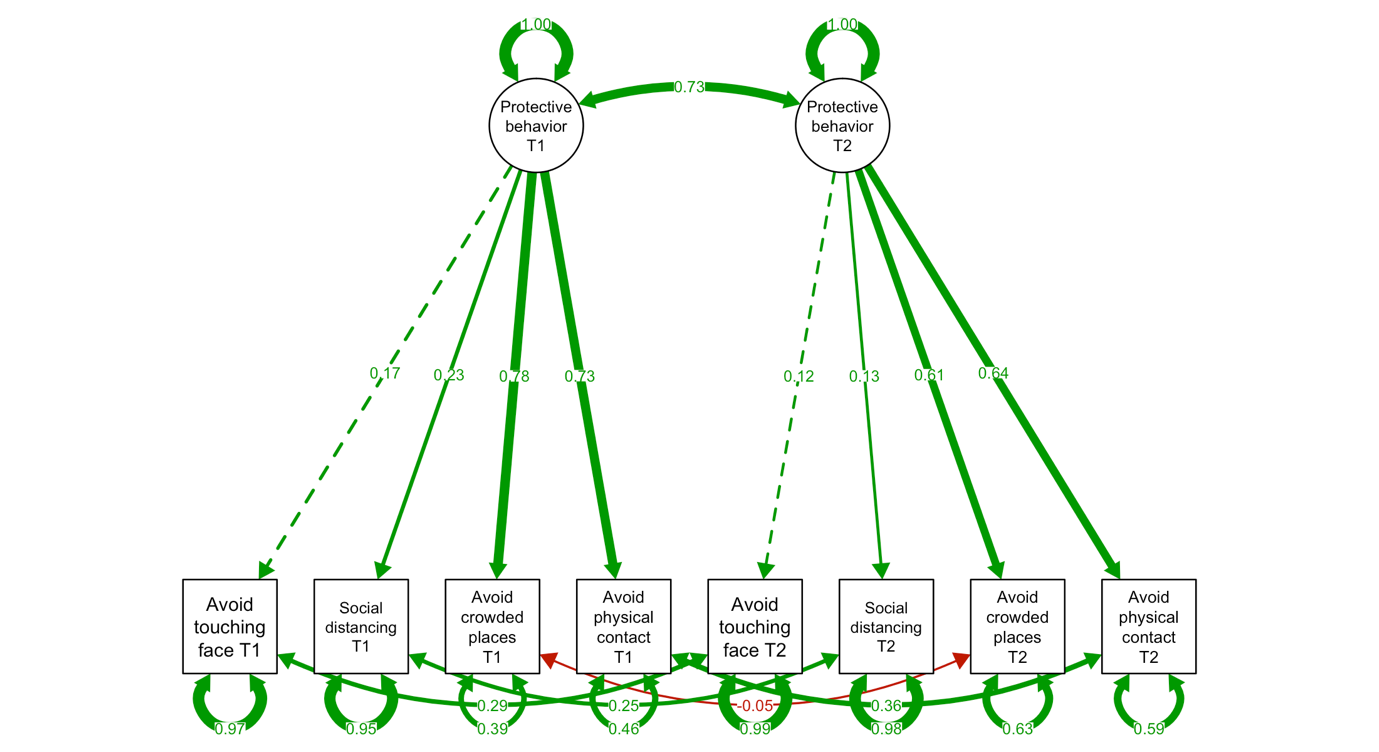


*Note.* The straight lines represent regression paths with the standardized parameter estimate reported. The double headed arrow curved lines pointing to the same variable represent residuals while those pointing to different variables represent residual covariance.

**References**

1. Rosseel Y. Lavaan: An R package for structural equation modeling. Journal of Statistical Software. 2012.

2. Hair JF, Black WC, Babin BJ, Anderson RE. Multivariate Data Analysis. 2010.

3. Hu LT, Bentler PM. Cutoff criteria for fit indexes in covariance structure analysis: Conventional criteria versus new alternatives. Structural Equation Modeling. 1999;6(1):1-55.
