# Supplementary figures and images for: Older adults’ perceptions of government handling of COVID-19: Predictors of protective behaviors from lockdown to post-lockdown
Source: PLoS One. 2022 Feb 2;17(2):e0263039. doi: 10.1371/journal.pone.0263039 (PMC8809562; doi:10.1371/journal.pone.0263039)

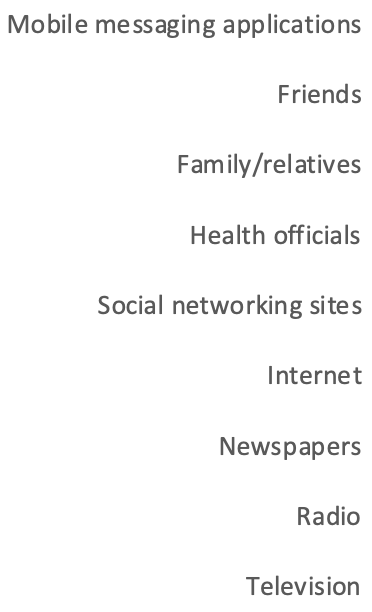

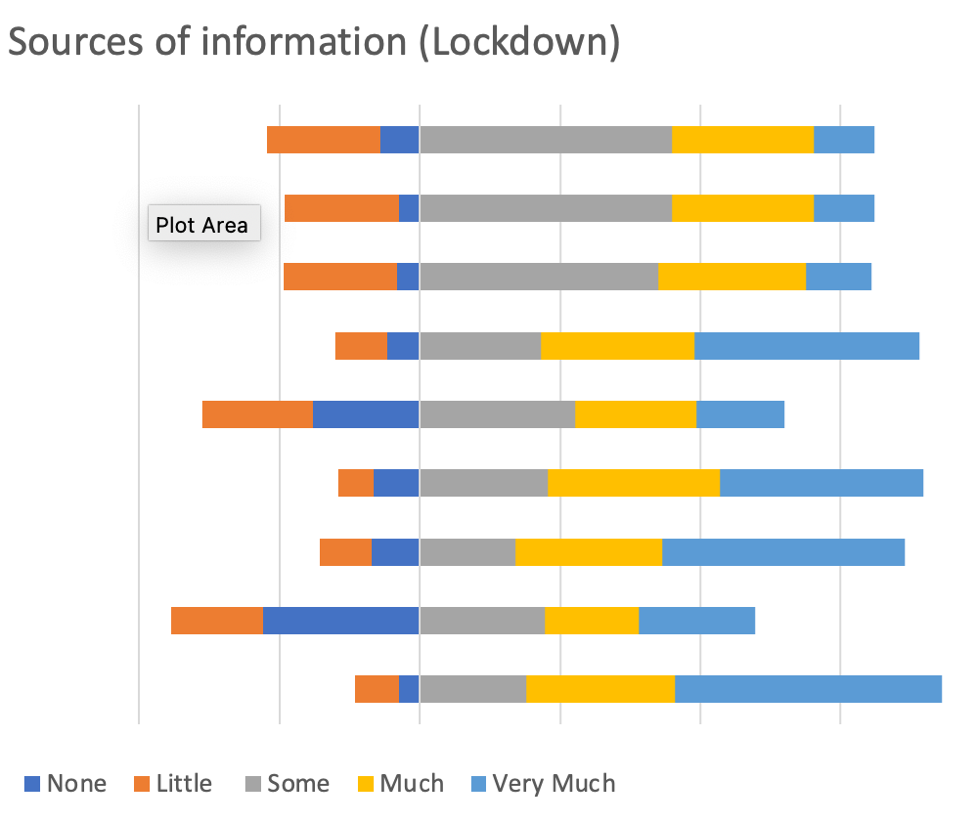


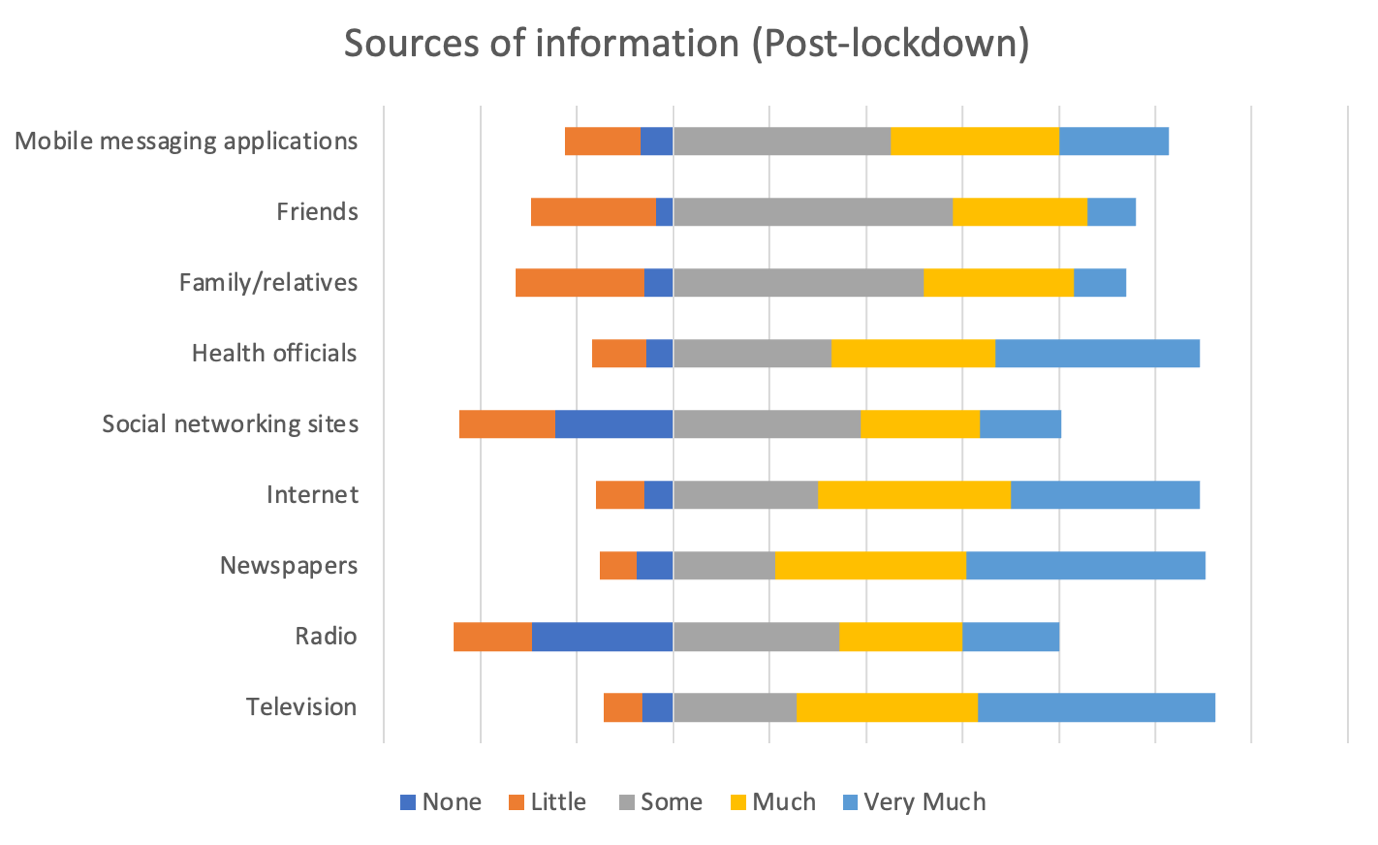

Supplement: S1 Table — Note. Participants were asked “Please indicate how much information about COVD-19 you have received from the following sources by selecting the appropriate option.” on a 5-point scale, “none”, “little”, “some”, “much”, and “very much”. (DOCX) [file pone.0263039.s004.docx]
